# The intricate interplay between microglia and adult neurogenesis in Alzheimer’s disease

**DOI:** 10.3389/fncel.2024.1456253

**Published:** 2024-09-18

**Authors:** Iris Früholz, Melanie Meyer-Luehmann

**Affiliations:** ^1^Department of Neurology, Medical Center ˗ University of Freiburg, Freiburg, Germany; ^2^Faculty of Medicine, University of Freiburg, Freiburg, Germany; ^3^Faculty of Biology, University of Freiburg, Freiburg, Germany

**Keywords:** Alzheimer’s disease, amyloid plaques, microglia, adult neurogenesis, neurodegeneration

## Abstract

Microglia, the resident immune cells of the central nervous system, play a crucial role in regulating adult neurogenesis and contribute significantly to the pathogenesis of Alzheimer’s disease (AD). Under physiological conditions, microglia support and modulate neurogenesis through the secretion of neurotrophic factors, phagocytosis of apoptotic cells, and synaptic pruning, thereby promoting the proliferation, differentiation, and survival of neural progenitor cells (NPCs). However, in AD, microglial function becomes dysregulated, leading to chronic neuroinflammation and impaired neurogenesis. This review explores the intricate interplay between microglia and adult neurogenesis in health and AD, synthesizing recent findings to provide a comprehensive overview of the current understanding of microglia-mediated regulation of adult neurogenesis. Furthermore, it highlights the potential of microglia-targeted therapies to modulate neurogenesis and offers insights into potential avenues for developing novel therapeutic interventions.

## 1 Microglia and adult neurogenesis

Microglia are the resident immune cells of the central nervous system (CNS), originating from primitive myeloid progenitors of the yolk sac, that migrate into the CNS during early embryonic development (Ginhoux et al., 2010; Kierdorf et al., 2013). Within the brain, these immature macrophages proliferate and differentiate into microglia, maintaining themselves exclusively through self-renewal under normal physiological conditions (Ginhoux et al., 2013). Traditionally viewed as the brain’s innate immune cells, microglia monitor the brain microenvironment and adopt a reactive inflammatory phenotype under harmful stimuli or pathological conditions (Ransohoff and Perry, 2009; Prinz and Priller, 2014; Meyer-Luehmann and Prinz, 2015), our understanding of microglia has expanded greatly in the last decade.

It is now firmly established that new neurons continue to be generated post-developmentally in specific neurogenic brain regions, namely the subventricular zone (SVZ) of the lateral ventricles and subgranular zone (SGZ) of the dentate gyrus in the hippocampus throughout adulthood in the mammalian brain, a process called adult neurogenesis (Altman and Das, 1965; Altman, 1969; Gage, 2000). The process of generating new neurons consists of several phases including proliferation, migration, differentiation, and survival of neurons. Neurogenesis originates from neural stem cells (NSCs) that give rise to neural progenitor cells (NPCs), which generate neuroblasts that differentiate into functional neurons and integrate into existing neural circuits (for review see Ming and Song, 2011). In the SGZ of the hippocampus, immature neurons migrate into the granule cell layer and differentiate into dentate granule cells, where they are functionally integrated into the hippocampal circuitry (van Praag et al., 2002), while cells in the SVZ migrate through the rostral migratory stream to the olfactory bulb (Gage, 2000).

Microglia are an essential component of the neurogenic niche, as they appear densely populated in the SVZ (Mosher et al., 2012), and exhibit a different heterogeneity, as microglia activation differs in neurogenic regions compared to non-neurogenic brain regions (Goings et al., 2006). Emerging evidence shows a fundamental role of microglia in various stages of adult neurogenesis under physiological conditions, including neuronal proliferation, differentiation, migration, survival and integration of newborn neurons into the pre-existing neuronal network (Aarum et al., 2003; Monje et al., 2003; Butovsky et al., 2006; Ziv et al., 2006; Choi et al., 2008; Sierra et al., 2010; Bachstetter et al., 2011; Vukovic et al., 2012; Reshef et al., 2014). Despite recognizing microglia’s influential role in neurogenesis, the field is only beginning to elucidate the intricate cellular and molecular interplay that facilitate this regulation.

Depletion of microglia in the adult mouse brain impaired the survival and migration of newly generated neuroblasts in the adult hippocampus and SVZ (Ribeiro Xavier et al., 2015; Kreisel et al., 2019). In contradiction, Kyle et al. (2019) found no involvement of microglia in adult SVZ neurogenesis. However, microglial ablation was further shown to reduce dendritic spine elimination, spine density and disrupt normal functional development of adult-born neurons in the olfactory bulb (Reshef et al., 2017).

The pruning of newly generated cells during their initial period of survival is a key mechanism by which microglia modulate hippocampal neurogenesis. Markedly, only small fractions of generated cells survive and join the hippocampal circuitry as mature neurons (Kempermann et al., 2003). Sierra et al. (2010) demonstrated that ramified microglia have an important function in effectively engulfing and clearing apoptotic cells through phagocytosis during the first days of their life in an immunologically silent process (i.e., without inflammation). Additionally, microglia have been reported to prune weak synaptic neuronal connections on mature neurons by phagocytosis, refining neural circuits and supporting functional integration of adult-born neurons (Stevens et al., 2007; Sierra et al., 2010; Tremblay et al., 2011; Schafer et al., 2012).

Through the secretion of various soluble factors, including cytokines, trophic factors, and growth factors, microglia support and modulate neurogenesis (Elkabes et al., 1996; Butovsky et al., 2006; Shigemoto-Mogami et al., 2014). Several studies have proposed that microglia-derived factors influence neurogenesis through various mechanisms, including: (1) determining the neuronal phenotype through instructive signaling, (2) promoting neural progenitor proliferation via secreted neurotrophic factors, and (3) regulating the survival and circuit integration of newborn neurons through the production of specific factors (Gemma and Bachstetter, 2013). Several *in vitro* studies show that microglia can guide precursor cell differentiation toward a neuronal phenotype, and support NPC migration and neuronal survival (Aarum et al., 2003; Morgan et al., 2004; Walton et al., 2006), through secreted factors like IGF-1 and BDNF. These factors aid NSC proliferation, NPC differentiation, migration, and newborn cell survival (O’Kusky et al., 2000; Aberg et al., 2003; Morgan et al., 2004; Scharfman et al., 2005; Choi et al., 2008; Li et al., 2008; Yuan et al., 2015). Co-culturing NPCs with microglia stimulated by IL-4, IL-10 or TGF-β increases new cell survival, proliferation, and/or neuronal differentiation (Battista et al., 2006; Butovsky et al., 2006; Kiyota et al., 2012; Matsui and Mori, 2018). Furthermore, pharmacological suppression of microglial activation inhibited neurogenesis and oligodendrogenesis by decreasing pro-inflammatory mediators like IL-1β, TNF-α, and IFN-γ (Shigemoto-Mogami et al., 2014) in a concentration-dependent manner (Butovsky et al., 2006; Bernardino et al., 2008).

Physical exercise and environmental enrichment (EE) can stimulate hippocampal neurogenesis (Kempermann et al., 1997; van Praag et al., 1999; Brown et al., 2003), potentially by modulating microglial activation and promoting a pro-neurogenic phenotype (Vukovic et al., 2012). This effect may involve increased expression of neurotrophic factors like BDNF and IGF-1 by microglia in the dentate gyrus (Kohman et al., 2012; Littlefield et al., 2015). However, some studies have reported conflicting results regarding the correlation between microglia and exercise-induced neurogenesis (Olah et al., 2009; Gebara et al., 2013).

The microglia–neuron crosstalk mediated by the CX3CL1-CX3CR1 signaling pathway has been critically implicated in regulating adult neurogenesis (reviewed in Al-Onaizi et al., 2020). Disruption of this pathway by genetic deletion or pharmacological antagonism of CX3CR1 in young adult rodents suppressed hippocampal neurogenesis, impaired synaptic integration, changed neuronal morphology and functionality (Bolós et al., 2018) and impaired cognitive functions and long-term potentiation (LTP) induction, potentially through the involvement of IL-1β (Bachstetter et al., 2011; Rogers et al., 2011; Vukovic et al., 2012; Reshef et al., 2014). Further, the aforementioned exercise-induced NPC proliferation changes in microglia are dependent on CX3CL1–CX3CR1 signaling (Vukovic et al., 2012).

## 2 Microglia dysregulation in Alzheimer’s disease

Alzheimer’s disease (AD) is a complex neurodegenerative disorder characterized by progressive cognitive decline. The neuropathological hallmarks of AD are extracellular amyloid-β (Aβ) plaques and intraneuronal neurofibrillary tangles (NFTs) composed of hyperphosphorylated tau protein (pTau), culminating in synaptic dysfunction, neuronal loss and brain atrophy (Duyckaerts et al., 2009; Selkoe, 2011). Aβ plaques generally appear early in disease before clinical symptoms occur, prompting the widely articulated “amyloid cascade hypothesis” (Hardy and Higgins, 1992). The imbalance between Aβ production and clearance, resulting in increased extracellular Aβ plaque deposition, is believed to be the principal pathogenic mechanism (Selkoe and Hardy, 2016).

Recent research has implicated microglia as key cellular players modulating neuroinflammation, neurodegeneration, and cognitive deficits in AD (Muzio et al., 2021). Loss of microglial homeostatic functions may significantly impact disease progression. Genome-wide association studies (GWAS) identified most AD risk genes being highly or exclusively expressed in microglia, suggesting microglial dysfunction contributes to AD development (Lambert et al., 2013; Jones et al., 2015; Villegas-Llerena et al., 2016; Wightman et al., 2021). These include genes implicated in immune functions like APOE, TREM2, PLD3, CD33, and others involved in the innate immune response, phagocytosis, and lipid metabolism (Efthymiou and Goate, 2017; Hansen et al., 2017; Verheijen and Sleegers, 2018).

During homeostasis, ramified microglia dynamically extend and retract processes to monitor the brain parenchyma (Nimmerjahn et al., 2005). Upon insults, microglia become activated, undergoing complex morphological and functional transformations to maintain homeostasis. Single-cell transcriptomic analyses revealed remarkable microglial heterogeneity in AD, challenging the traditional dichotomous classification of M1 (pro-inflammatory) and M2 (anti-inflammatory) phenotypes (Keren-Shaul et al., 2017; Friedman et al., 2018; Gerrits et al., 2021). Notably, a distinct microglial subpopulation termed “disease-associated microglia” (DAM) has been identified in both mouse models and human AD brains, exhibiting a unique transcriptional signature (Keren-Shaul et al., 2017; Mathys et al., 2017). Additionally, “dark microglia” or “microglial neurodegenerative” (MGnD) phenotypes were described (Bisht et al., 2016; Krasemann et al., 2017) that associate with Aβ plaques and show decreased expressions of homeostatic microglia marker genes.

This microglial association with dense-core Aβ plaques, but not diffuse plaques, has been observed in both AD patients and transgenic mouse models (Perlmutter et al., 1990; Frautschy et al., 1998; Stalder et al., 1999; Meyer-Luehmann et al., 2008). Microglia possess a range of pattern recognition receptors (PRRs; Khoury et al., 1996; Venegas and Heneka, 2017) that detect Aβ, thereby influencing the microglial phenotype and triggering an inflammatory response (Salminen et al., 2009). Microglia activation induces morphological alterations and functional remodeling, transitioning from a ramified to an amoeboid, activated state (Kettenmann et al., 2011). In response to lesions or infections, reactive microglia migrate to the affected areas and undergo mitotic proliferation (Kettenmann et al., 2011; Heneka et al., 2015). Additionally, microglia can initiate receptor-mediated uptake and lysosomal degradation of Aβ, as well as produce various Aβ-degrading enzymes (Heneka, 2017). Aβ stimulates a pathway dependent on nuclear factor-kappa B (NF-B), which subsequently activates the production of inflammatory mediators and proinflammatory cytokine secretion, including IL-1β, TNF-α, and IL-6, as well as ROS and NO (Combs et al., 2001; Walker et al., 2001; Ho et al., 2005). Post-mortem brain tissues from patients suffering from AD show increased production of pro-inflammatory cytokines, particularly in the vicinity of amyloid plaques (Griffin et al., 1995).

One of the primary microglial functions in AD is Aβ clearance through phagocytosis (Hickman et al., 2008). The microglial surface receptor TREM2 is critical for this process, as it binds to Aβ-lipoprotein complexes (Wang et al., 2015) and promotes the conversion of microglia to the DAM phenotype, responsible for Aβ phagocytosis (Keren-Shaul et al., 2017). Loss of TREM2 impairs Aβ phagocytosis, leading to increased amyloid seeding in AD mouse models (Wang et al., 2015; Parhizkar et al., 2019). This clearance capacity appears impaired in later stages of AD, potentially due to age-related alterations or the overwhelming accumulation of Aβ (El Khoury et al., 2007; Hickman et al., 2008). Notably, microglia also form a protective barrier around Aβ deposits, compacting amyloid fibrils into a tightly packed core and potentially limiting neurotoxicity. This “corralling” function appears to be more effective for small, early-stage plaques and is compromised in the absence of TREM2 (Condello et al., 2015; Yuan et al., 2016). Furthermore, TREM2 has been implicated in controlling the physiological process of microglia-mediated synapse elimination during synaptic pruning (Filipello et al., 2018; Tagliatti et al., 2024). It enhances microglial phagocytic efficiency in clearing damaged synapses around Aβ plaques (Fracassi et al., 2023), by activating signal transduction pathways that promote microglial chemotaxis, phagocytosis, and survival (Konishi and Kiyama, 2018), offering protection against Aβ toxicity and limiting spreading of damage in AD models (Jiang et al., 2018; Lee et al., 2018).

The neuron–microglia signaling unit involving fractalkine (CX3CL1) and its receptor CX3CR1 regulates microglial inflammation in neurodegenerative diseases (Cardona et al., 2006; Bhaskar et al., 2010). CX3CR1 deficiency reduced Aβ load but worsened neuronal and behavioral deficits in a plaque-independent manner in AD mice (Bhaskar et al., 2010; Lee et al., 2010). Furthermore, depletion with CSF1R inhibitor reduced Aβ plaque compaction and increased diffuse plaques and dystrophic neurites in AD mouse models (Spangenberg et al., 2016; Casali et al., 2020). Interestingly, microglia may also contribute to Aβ propagation of amyloid pathology by facilitating the spreading of Aβ in an CX3CR1 dependent manner (d’Errico et al., 2022). These findings highlight the complex role of microglia and fractalkine signaling in the pathogenesis of AD.

Microglia exhibit a double-edged role in AD pathogenesis. Initially, they exert neuroprotective functions by clearing Aβ protein aggregates, producing neurotrophic factors, and forming physical barriers around plaques. These functions reduce neurotoxicity, limit plaque growth, while simultaneously delaying disease progression and onset of AD symptoms. As AD progresses, sustained microglial activation induced by Aβ leads to a detrimental pro-inflammatory state. Growing evidence indicates that chronic neuroinflammation, mediated by activated microglia secreting neurotoxic cytokines and inflammatory mediators, causes a decline in microglial homeostatic functions (Sarlus and Heneka, 2017), synaptic toxicity and consequently synapse loss and neuronal injury (Hong et al., 2016). Microglia are critically involved in parenchymal and vascular plaque formation, growth, and compaction, as well as influencing neuronal gene expression and mitigating neuritic dystrophy. Their ability to internalize and deposit aggregated Aβ is vital for the initial plaque development and progression, highlighting their complex involvement in AD (Spangenberg et al., 2019). Furthermore, microglia can promote Aβ pathology by stimulating neurons to overproduce Aβ, leading to a self-perpetuating cycle of neuroinflammation and neurodegeneration. Moreover, chronic stimulation of microglia by pathological Aβ deposits can cause these initially protective microglia to transform into a dysfunctional phenotype, thereby further aggravating AD progression.

Importantly, age is a critical factor influencing microglial function in AD. Senescence in microglia is characterized by changes in density, activation state, morphology, lipofuscin accumulation, phenotype, cytokine expression, and phagocytic capacity (Perry et al., 1993; Hart et al., 2012; Li et al., 2023; Stillman et al., 2023), contributing to persistent inflammation and Aβ pathology (Michelucci et al., 2009).

Taken together, microglia exhibit remarkable diversity and adaptability. Unraveling microglia’s multifaceted roles, both in health and across AD progression, is crucial for understanding the disease’s underlying mechanisms.

## 3 Interplay between microglia, adult neurogenesis and Alzheimer’s disease

Our comprehension of the role of adult neurogenesis in learning and memory among healthy individuals is still evolving, and we have yet to fully understand how impaired neurogenesis contributes to cognitive decline in aging and AD. The dentate gyrus of the hippocampus, a brain region critical for pattern separation, emotional memory, cognitive flexibility, learning and memory formation, exhibits adult neurogenesis (Lazarov and Hollands, 2016).

In AD, the hippocampus is among the earliest and most severely affected brain regions (Selkoe, 2011), with progressive memory impairment, associated with hippocampal degeneration (Thompson et al., 2004). Recent evidence suggests that impaired adult neurogenesis in the SVZ and SGZ, caused by intracellular accumulation of Aβ oligomers, may constitute an early event in AD pathogenesis, actively contributing to disease progression (Scopa et al., 2020). However, whether Aβ directly impairs neurogenesis and the hippocampal circuitry involved in memory formation, or whether altered neurogenesis is a byproduct of AD pathology that contributes insignificantly to the AD phenotype and cognitive dysfunction, remains debated (Hollands et al., 2016; Li Puma et al., 2021). Considerable discrepancies exist regarding whether neurogenesis is enhanced or repressed in AD (Mu and Gage, 2011; Sierra et al., 2011), likely stemming from varying approaches, markers, tissue preparation, and disease progression effects on neurogenesis, or AD’s impact on neuronal maturation stages. A valid hypothesis is that the early overstimulation of neurogenesis as a compensatory mechanism by Aβ causes a depletion of the stem cell pool and a decline in neurogenesis. Over time, as senile plaques form, the balance may shift toward neurotoxic, fibrillar Aβ, which further exacerbates the neurogenesis rate (Kent et al., 2020). More recent studies have confirmed impaired adult hippocampal neurogenesis (AHN) in AD and cognitively impaired patients (Moreno-Jiménez et al., 2019; Tobin et al., 2019), as detected by single-nucleus RNA sequencing (snRNA-seq; Zhou et al., 2022). The number of NPCs, neuroblasts and adult-born neurons progressively declined with advancing Braak stages in AD patients, preceding tangle and plaque formation (Moreno-Jiménez et al., 2019).

Similar to human studies, contradictory conclusions from varying mouse models have hindered a clear understanding of neurogenesis in AD. Furthermore, the stage at which neurogenesis is impaired varies, with certain models showing defects exclusively during the maturation stage (Hollands et al., 2017). Comprehensive summaries of the results can be found elsewhere (Babcock et al., 2021; Choi and Tanzi, 2023). Briefly, increased neurogenesis was exhibited in APPsw transgenic (Jin et al., 2004) and APP23 mice (Ermini et al., 2008), while presenilin-1 (PS-1) overexpression models and APP/PS-1 double transgenic mice exhibited varying neurogenesis patterns, with both increases and decreases depending on age and disease progression (Chevallier et al., 2005; Sotthibundhu et al., 2009; Demars et al., 2010; Biscaro et al., 2012; Zeng et al., 2016). However, mounting evidence from studies including 3xTg (Rodríguez et al., 2008; Hamilton et al., 2010), 5xFAD (Moon et al., 2014; Ziegler-Waldkirch et al., 2018b), and several APP overexpression models, supports alterations in AHN. *In vivo* and *in vitro* studies showed Aβ contribute to AHN impairment in AD (for review Culig et al., 2022; Farioli-Vecchioli et al., 2022).

Severe impairment of AHN and/or maturation of newborn neurons in early AD stages, occurring before memory impairment, has been observed. Interestingly, stimulating neurogenesis rescues cognitive deficits (Choi et al., 2018; Ziegler-Waldkirch et al., 2018b), suggesting impaired adult neurogenesis might contribute to AD-associated cognitive dysfunction.

Ablation of adult neurogenesis in AD transgenic mice substantially reduced the number of adult-born neurons and induced cognitive deficits by compromising hippocampal functions (Hollands et al., 2017; Choi et al., 2018; Zhang et al., 2021). Conversely, stimulating neurogenesis at the dentate gyrus through genetic, pharmacological, EE, or physical exercise rescues cognitive deficits in transgenic 5 × FAD and 3xTg mice, reduces amyloid burden (Valero et al., 2011; Sun et al., 2018; Ziegler-Waldkirch et al., 2018b; Kim et al., 2019), and simultaneously increases BDNF levels (Choi et al., 2018). Finally, EE increases progenitor proliferation, survival, differentiation, and dendritic arborization (Mirochnic et al., 2009; Valero et al., 2011).

Given microglia’s crucial role in regulating adult neurogenesis and their involvement in the inflammatory response associated with neurodegenerative diseases, a pertinent question arises: How do microglia modulate adult neurogenesis in the context of AD? Microglia are activated in the AD brain, causing inflammation and altering their effects on neurogenesis. During chronic stress, aging, and neurodegenerative diseases, microglia exhibit a pro-inflammatory phenotype, which can compromise the neurogenic cascade by releasing neurotoxic pro-inflammatory cytokines like IL-1β, IL-6, IL-17, and TNFα (Vallières et al., 2002; Zou and Crews, 2012; Wu et al., 2013; Liu et al., 2014). Evidence links microglia to facilitating AD pathology, as IGF1 expression, implicated in regulating neurogenesis, increases in APP/PS1 mice along with increased microglial activation and reduced SGZ neurogenesis (Myhre et al., 2019). Furthermore, reducing TGFβ in another AD mouse model accelerated neurodegeneration and AD-like pathology (Tesseur et al., 2006). Administration of minocycline, a microglia activation inhibitor, improved hippocampal-dependent learning and increased dentate granule cell survival in APP/PS1 mice, concomitantly decreasing inflammatory cytokines and microglial cells. Hence, the activated and inflammatory microglial phenotype causes neurogenetic and cognitive decay. However, Aβ levels or Aβ-related morphological deficits remained unaffected (Biscaro et al., 2012). Repopulation of microglia after CSF1R inhibitor cessation reverses AD-associated cognitive deficits, dysregulated neurotrophic signaling pathway and hippocampal neurogenesis by restoring BDNF expression in microglia in 5xFAD mice (Wang et al., 2023).

In parallel, physical exercise attenuates the age-dependent aberrant microglia activation in an AD model (Nichol et al., 2008) and reduced hyper-activated microglia in aged mice, increasing their pro-neurogenic phenotype by upregulating IGF1 (Kohman et al., 2012). Environmental enrichment reduced Aβ plaque load by activating phagocytic microglia in 5xFAD mice, reviving adult hippocampal neurogenesis and rescuing cognitive deficits (Ziegler-Waldkirch et al., 2018a,b). Additionally, EE reversed Aβ seeding-induced olfactory deficits in the olfactory bulb of 5xFAD mice (Ziegler-Waldkirch et al., 2022).

Notably, deficits in EE-dependent AHN observed in an AD model mouse expressing PS1 were completely restored upon microglia depletion via CSF1R antagonist (Ortega-Martinez et al., 2019). Impairment of CX3CR1 in AD disrupts hippocampal neurogenesis and learning due to increased IL-1β (Parkitny and Maletic-Savatic, 2021). In this regard, CX3CR1 depletion in AD models has been shown to mitigate AD-associated pathology by enhancing microglial phagocytosis (Lee et al., 2010; Liu et al., 2010). Amyloid pathology accelerates microglial senescence, progressively impairing their phagocytic capacity as degeneration increases (Flanary et al., 2007; Miller and Streit, 2007). Consequently, as microglial phagocytosis maintains the neurogenic niche, diminished phagocytic capacity of senescent microglia likely dysregulates the niche environment, suppressing AHN due to impaired clearance of Aβ and other factors disrupting niche homeostasis.

Of note, during physiological aging, both rodents and humans experience a significant decline in adult neurogenesis within the neurogenic niches (Kuhn et al., 1996; Kase et al., 2020). In addition, microglia undergo transcriptional and morphological changes with age, adopting a pro-inflammatory phenotype that is associated with impairments in neurogenesis and synaptic plasticity (Walton et al., 2006; Kohman et al., 2012; Vukovic et al., 2012; Gebara et al., 2013; Solano Fonseca et al., 2016). Studies have shown that microglia in aged mice, when depleted, promote the proliferation and maturation of NSC, indicating a potential anti-neurogenic role of microglia in the aging brain (Vukovic et al., 2012; Elmore et al., 2018). Thus, age-related alterations in microglial signatures may have additive derogatory effects on neurogenesis in the aged AD brain, exacerbating the decline in brain function over time.

**Figure 1:**
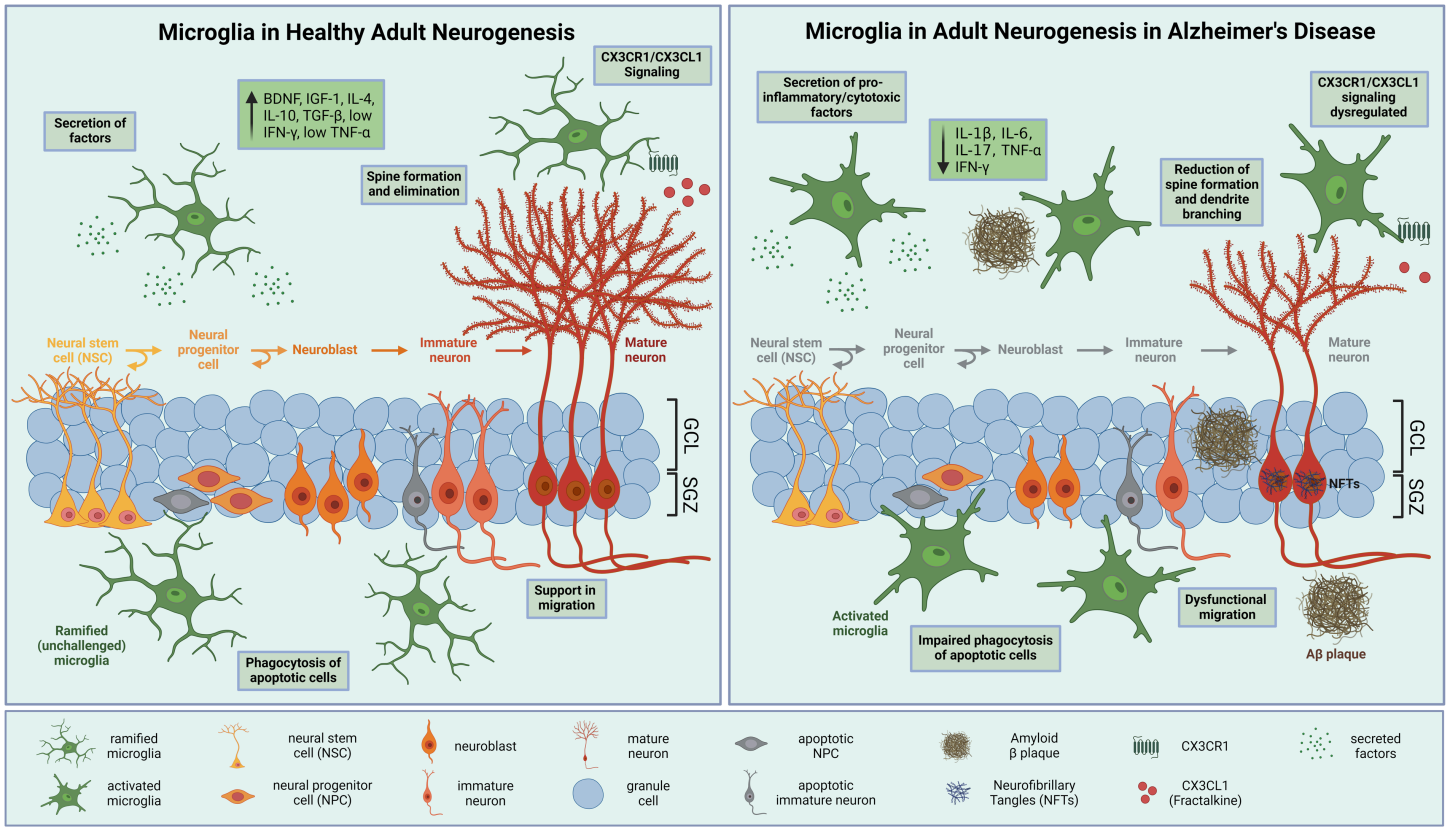
Microglial modulation of hippocampal neurogenesis in health and disease. **(A)** During physiological conditions, neural stem cells (NSCs) in the hippocampal subgranular zone (SGZ) produce neural progenitor cells (NPCs), which differentiate into neuroblasts and migrate into the granule cell layer (GCL), where the cells mature and integrate into the hippocampal neural circuitry. Ramified microglia effectively eliminate excess apoptotic newborn cells by phagocytosis. Secretion of neurotrophic factors effects proliferation, differentiation, and survival of neurons. In addition, microglia prune synapses and induce spine formation to support adult neurogenesis. Furthermore, microglia communicate with neurons through CXCR1/CX3CL1 signaling, which contribute to the ability of microglia to maintain a ramified phenotype. **(B)** In Alzheimer’s disease neurogenesis is reduced. Proliferation and differentiation of neurons are inhibited due to amyloid-β (Aβ) aggregation and neurofibrillary tangles (NFTs). Activated microglia adapt a pro-inflammatory phenotype releasing neurotoxic cytokines that impair neurogenesis and synaptic integrity, such as interleukin (IL)-1β, IL-6, IL-17 and tumor necrosis factor (TNF)-α. These cytokines have profound detrimental effects on adult neurogenesis by reducing proliferation, differentiation, survival, and integration of newborn neurons. Microglia display an impaired phagocytic activity and CX3CR1-CX3CL1 signaling. BDNF, brain-derived neurotrophic factor; IGF-1, insulin-like growth factor 1; IL-(1β,4,6,10,17), Interleukin-(1β,4,6,10,17); TGFβ, transforming growth factor β; IFN-γ, interferon-γ; TNF-α, tumor necrosis factor α. Created with BioRender.com.

In summary, activated microglia in AD impair multiple processes of neurogenesis (Figure 1). Further studies are needed to elucidate how microglial control of neurogenesis is influenced by AD pathological hallmarks.

## 4 Therapeutic targeting of microglia in Alzheimer’s disease

Malfunctioning of adult neurogenesis is considered a contributing factor to neurodegenerative diseases like AD, potentially leading to cognitive decline. Restoring or stimulating neurogenesis by increasing NSC proliferation in AD patients or individuals at high risk could provide a potential approach to prevent, delay, or counteract disease progression in the early stages of disease, particularly in terms of learning and memory impairments. Furthermore, interventions that improve or stimulate endogenous neurogenesis have been shown to decrease AD hallmarks, like Aβ accumulation and pTau. This indicates a potential bidirectional relationship: while Aβ and pTau impact neurogenesis, the molecular pathways governing neurogenesis may also influence Aβ clearance and tau phosphorylation (Lazarov et al., 2005; Biscaro et al., 2009; Choi et al., 2018; Ziegler-Waldkirch et al., 2018b; Kim et al., 2019). However, this strategy presents several crucial considerations. Highly promoted neurogenesis can deplete NSCs and cause early cessation of neurogenesis (Encinas et al., 2011; Sierra et al., 2015). Instead of boosting neuron production and proliferation in an already aged and Alzheimer’s-diseased brain, it may prove more beneficial to devise strategies aimed at preserving existing neurons and NPCs in presymptomatic individuals before age-related neuronal loss occurs. Additionally, a major challenge is the poor long-term survival of new neurons, likely due to the pathological and inflammatory environment. Therefore, exploring how microglia in different activation states regulate adult neurogenesis is highly warranted. Modulating microglial function and phenotype represents a promising approach to enhance adult neurogenesis and potentially alleviate cognitive deficits in AD. Restoring the homeostatic, neuroprotective microglial phenotype could normalize defective neurogenesis. Specific microglial subpopulations in the SVZ have been shown to promote adult neurogenesis, suggesting that targeting these populations could be therapeutically beneficial in AD (Ribeiro Xavier et al., 2015). Anti-inflammatory approaches skewing microglia toward an alternatively-activated, pro-neurogenic phenotype could enhance endogenous neurogenesis and potentially delay neurodegeneration (Varnum and Ikezu, 2012; Shohayeb et al., 2018; Muzio et al., 2021). For example, stimulating fractalkine CX3CL1/CX3CR1 signaling reduces neuroinflammation and may protect against age or disease-related neurogenic decline (Bachstetter et al., 2011). Furthermore, enhancing the neuroprotective, phagocytic phenotype of microglia could help clear pathological protein aggregates and reduce neuroinflammation. Clinical trials are ongoing to test microglia-targeted therapies like the CSF1R kinase inhibitors in AD (Martin-Estebane and Gomez-Nicola, 2020). Inhibiting CSF1R modulates microglial activation and has shown promising outcomes in animal models (Dagher et al., 2015; Spangenberg et al., 2016, 2019), particularly in regard to neurogenesis (Elmore et al., 2018). Genetic engineering of microglia is an emerging strategy being explored, including altering gene expression to reprogram microglia into a neuroprotective state for targeted delivery of therapeutics (Luo and Sugimura, 2024). However, technical limitations have prevented clinical translation so far. In summary, while still in early stages, modulating microglial phenotype and function through various approaches like small molecules, genetic engineering, or immunomodulation represents a promising avenue for developing novel neurorestorative therapies targeting neuroinflammation and neurogenesis deficits in neurodegenerative diseases like AD.
